# POU2F2 promotes the proliferation and motility of lung cancer cells by activating AGO1

**DOI:** 10.1186/s12890-021-01476-9

**Published:** 2021-04-08

**Authors:** Ronggang Luo, Yi Zhuo, Quan Du, Rendong Xiao

**Affiliations:** Department of Thoracic Surgery, The First Affiliated Hospital of Fujian Medical University, No.20 Chazhong Road, Fuzhou City, 350005 Fujian Province China

**Keywords:** POU2F2, Lung cancer, Proliferation, Motility AGO1, Therapeutic target

## Abstract

**Background:**

To detect and investigate the expression of POU domain class 2 transcription factor 2 (POU2F2) in human lung cancer tissues, its role in lung cancer progression, and the potential mechanisms.

**Methods:**

Immunohistochemical (IHC) assays were conducted to assess the expression of POU2F2 in human lung cancer tissues. Immunoblot assays were performed to assess the expression levels of POU2F2 in human lung cancer tissues and cell lines. CCK-8, colony formation, and transwell-migration/invasion assays were conducted to detect the effects of POU2F2 and AGO1 on the proliferaion and motility of A549 and H1299 cells in vitro. CHIP and luciferase assays were performed for the mechanism study. A tumor xenotransplantation model was used to detect the effects of POU2F2 on tumor growth in vivo.

**Results:**

We found POU2F2 was highly expressed in human lung cancer tissues and cell lines, and associated with the lung cancer patients’ prognosis and clinical features. POU2F2 promoted the proliferation, and motility of lung cancer cells via targeting AGO1 in vitro. Additionally, POU2F2 promoted tumor growth of lung cancer cells via AGO1 in vivo.

**Conclusion:**

We found POU2F2 was highly expressed in lung cancer cells and confirmed the involvement of POU2F2 in lung cancer progression, and thought POU2F2 could act as a potential therapeutic target for lung cancer.

**Supplementary Information:**

The online version contains supplementary material available at 10.1186/s12890-021-01476-9.

## Background

Lung cancer often originates from bronchial mucosa or gland, and is a malignant tumor with relative high mortality [1, 2]. In 2018, the mortality of male lung cancer ranked the highest in the world, while lung cancer incidence rate was the highest among women, and the mortality rate was only second [3]. According to the histopathological features, lung cancer is mainly divided into small cell lung cancer and non-small cell lung cancer [4], and the latter is subdivided into squamous cell carcinoma, large cell carcinoma, and adenocarcinoma [5]. In view of the lack of significant early symptoms of lung cancer, patients are often in an advanced stage [6]. In recent years, the targeted therapy of lung cancer has made important progress, and a variety of targeted drugs emerge in endlessly [7, 8]. In order to further improve the prognosis of patients, it is still necessary to identify more potential therapeutic targets.

POU domain class 2 transcription factor 2 (POU2F2), also named octamer-binding protein 2 (OCT2), was a B-cell-restricted transcription factor belonged to POU domain family, which used the POU domain to bind DNA [9, 10]. It was reported that POU2F2 could bind to an octamer DNA motif of 5′-ATGCAAAT-3′ [11]. POU2F2 directed the B-cell-restricted expression of Ig genes through the bind of octamer motifs in the promoters [12]. Additionally, the POU2F2-knockout mice die shortly after birth [13]. Previous study indicated that the overexpression of POU2F2 could accelerate the fracture healing via WNT/beta-catenin pathway [14]. POU2F2 also regulated the distribution of dorsal interneurons in mice [15].

Recently, the critical role of POU2F2 in the progression of cancers has been revealed in depth [16]. POU2F2 was highly expressed in multiple types of tumor tissues, such as gastric cancer and liver cancer [16, 17]. POU2F2-oriented network could promote the metastasis of gastric cancer [16]. Immunohistochemical assays showed the upregulation of POU2F2 in systemic anaplastic large cell lymphomas [18]. Additionally, POU2F2 expression predicted the prognosis in patients with newly diagnosed acute myeloid leukemia [19]. However, the effects of POU2F2 on the progression of lung cancer is still unclear.

Herein, we found the high POU2F2 expression in human lung cancer tissues and cells. POU2F2 expression was correlated with patients’ prognosis. We also found POU2F2 could promote the proliferation, and motility of lung cancer cells via AGO1, and contribute to tumor growth of lung cancer cells in mice. We therefore thought POU2F2 could act as a promising target for lung cancer.

## Methods

### Antibodies, siRNAs, and plasmids

Anti-POU2F2 (1:200 for Immunohistochemical (IHC), 1:1000 for Immunoblot, and 1:50 for CHIP, ab179808, Abcam, UK), anti-AGO1 (1:100, ab129304, Abcam, UK), anti-Ki67 (1:1000, ab16667, Abcam, UK), anti-PCNA (1:1000, ab29, Abcam, UK), anti-N-cadherin (1:500, ab76011, Abcam, UK), and anti-β-actin (1:1000, ab8226, Abcam,UK). POU2F2 and AGO1 siRNAs were bought from Riobio Biotech. The shRNA of POU2F2, plv-vector, plv-POU2F2, plv-AGO1, and pGL3-Basic plasmids was purchased from Addgene plc.

### Human tissue samples and IHC assays

The lung cancer tissues and the adjacent tissues were all obtained from The Fujian Medical University. IHC assays were performed on these tissues. Resin was used for the embedding of tissues, and 5-µm-thick sections were fixed with the 4% paraformaldehyde (PFA) for half an hour at room temperature. Subsequently, sections were blocked with 2% BSA (Sigma-Aldrich; Merck) for half an hour at room temperature. Sections were treated with the antibody for 2 h at room temperature. Then the sections were treated with the secondary antibodies for 1.5 h, and the diaminobenzidine was used as a type of chromogen substrate. A light microscope was used for imaging at 100 × and 200 × magnification. All procedures performed in studies involving human participants were in accordance with the standards upheld by the Ethics Committee of the First Affiliated Hospital of Fujian Medical University and with those of the 1964 Helsinki Declaration and its later amendments for ethical research involving human subjects.

### Cell culture and transfection

4 different types of human lung cancer cell lines, calu-3, calu-6, A549, and H1299, and a normal bronchial epithelial cell line, named BEAS-2B, were bought from ATCC and maintained in dulbecco’s minimum essential medium (DMEM), supplemented with 10% of fetal bovine serum (FBS) and incubated at 37 °C in a 5% CO_2_ incubator.

The plasmids or siRNAs were transfected into lung cancer cells by lipofectamine 3000 (Invitrogen, US). The in vivo assays used Stable POU2F2 depletion cells were identifed by POU2F2 shRNA transfection.

### Immunoblot assay

Protein samples were isolated by SDS-PAGE, transferred onto the NC membranes, followed by 5% fat-free milk blocking. NC membranes were subsequently incubated with POU2F2, AGO1, and β-actin antibodies for 1.5 h. Subsequently the membranes were incubated with HRP-labeled secondary antibodies for an hour. Signals were detected by ECL kit (Novex ECL Reagent kit; Thermo Fisher, USA). These images were analyzed with the Image J (National Institutes of Health, USA).

### CCK-8 and colony formation assay

For CCK-8 assays, both A549 and H1299 cells (800 cells/well) were added into 96-well plates for 3 days. CCK-8 agent was added into the plate for 3 h at 37 ℃. Then the OD value in each well was detected at 490 nm wavelength.

For colony formation assays, cells were plated into 24-well plates with a density of nearly 100 cells perwell and maintained for 2 weeks. The colonies were fixed with methanol at – 20 ℃ for half an hour and stained with 0.1% crystal violet for half an hour. Colonies were then counted.

### Transwell-migration and transwell-invasion assays

Lung cancer cells were transfected, trypsinized, and re-suspended in medium without serum.

For transwell-invasion assay, the upper chambers of 8.0 µm membrane pore transwell filters coated with 20% matrigel (For transwell-migration assay without matrigel) and incubated at 37 °C for half an hour.

Then lung cancer cells in 150 µL medium were all added to the upper chambers to stimulate the migration toward the bottom containing complete medium. After 24 h, the remaining cells in the top chamber were removed, and the remaining lung cancer cells were fixed with 4% paraformaldehyde (PFA), followed by the staining of 0.1% crystal violet, and the OD value at 490 nm wavelength was measured.

### Tumor growth in vivo assay

To measure tumor volume, A549 cells stably transfected with the shRNA plasmids were injected subcutaneously into the nude mice. After 1 week, tumors were established, and the volume of tumors was measured every week and calculated. After 7 weeks, all tumors were isolated, and tumor growth curves were calculated respectively, and the weight of tumors were also measured. All animal experiments were approved by the Ethics Committee of the First Affiliated Hospital of Fujian Medical University.

### CHIP and luciferase assays

CHIP assays wereconducted by a CHIP assay kit (CST, USA). A total of 108 A549 cells were crosslinked, mixed, and lysed. Then the samples were sonicated to shear the DNA into between 600 and 800 bp. Chromation fraction was isolated by the anti-POU2F2 or IgG antibodies. Then, the mix was enriched by the agarose of protein A/G, which were subsequently isolated and washed. DNA was purified, and the qPCR assays were subsequently performed to detect site 1, site 2 and site 3 level.

For luciferase assays, A549 cells were transfected with plv-vector or plv-POU2F2 plasmids overnight. After 24 h, cells were lyzed, and the of AGO1 wild or mutant cell luciferase activities were detected by a luciferase assay kit.

### Statistics

In this study, graphpad 6.0 software was used for the statistical analysis. All data were represented as mean ± SEM. K-M survival analysis was used to assess the survival of patients. Student’s *t* test was for statistical comparisons, and *p* < 0.05 was considered significant. *indicated *p* < 0.05, **p < 0.01, and ^##^*p* < 0.001, respectively.

## Results

### POU2F2 was highly expressed in human lung cancer tissues and cell lines, and correlated with lung cancer patients’ prognosis

**Fig. 1:**
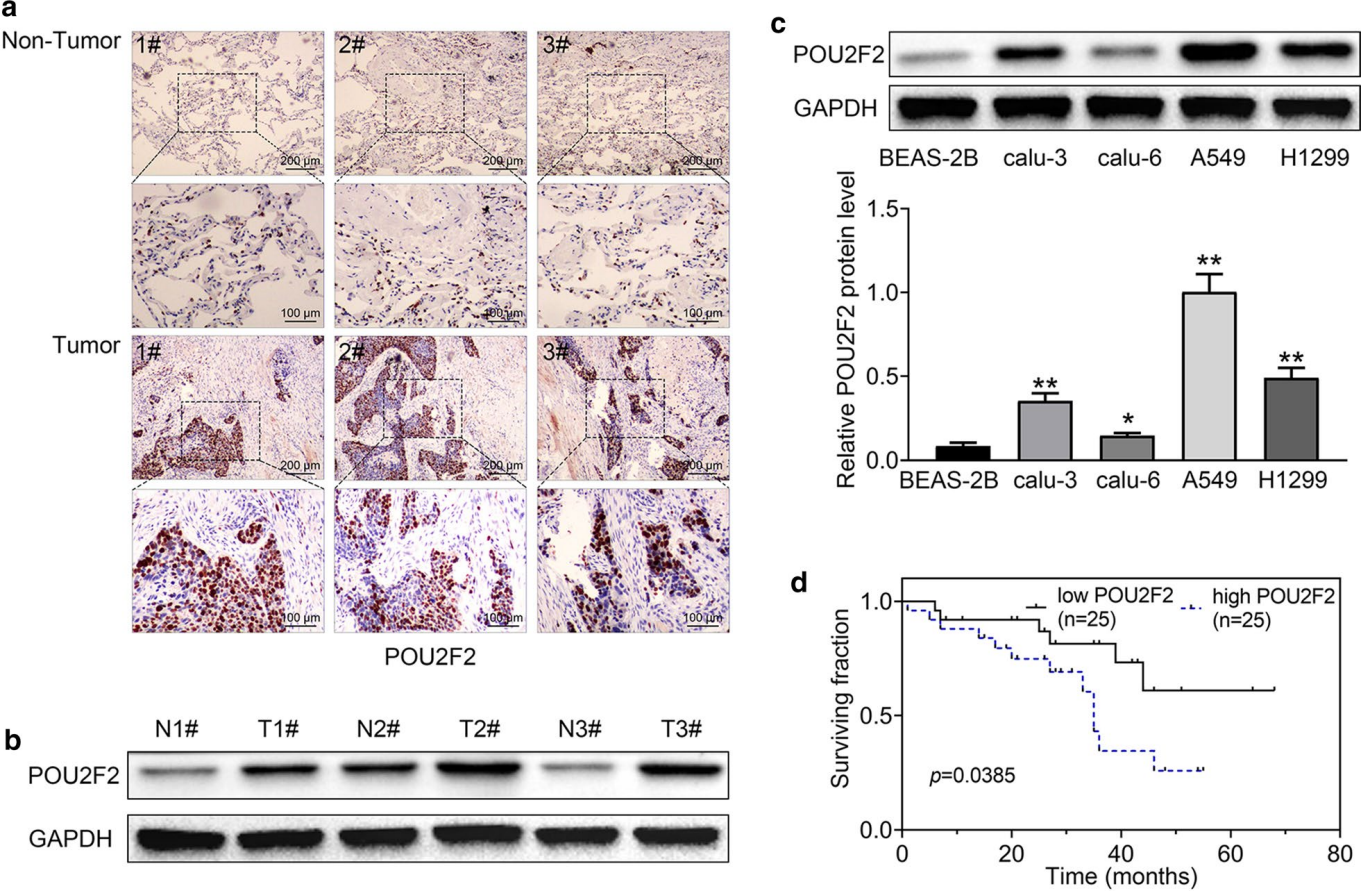
POU2F2 was highly expressed in human lung cancer tissues and cell lines, and correlated with the prognosis of lung cancer patients. **a** Representative images of POU2F2 expression level detected by Immunohistochemical (IHC) in lung cancer tissues and the corresponding normal tissues from three different patients (× 100 and × 200 magnification, respectively). **b** Immunoblot assays showed the expression levels of POU2F2 in lung cancer tissues and the corresponding normal tissues from three different patients. These images were analyzed with the Image J (National Institutes of Health, USA). Original image blots are presented in Additional file 1. **c** Immunoblot assays showed the expression levels of POU2F2 in 4 types of human lung cancer cell lines, including calu-3, calu-6, A549, and H1299, and a normal bronchial epithelial cell line BEAS-2B. These images were analyzed with the Image J (National Institutes of Health, USA). Original image blots are presented in Additional file 2. **d** Kaplan–Meier survival analysis was performed to analyze the correlation between POU2F2 expression and the survival fraction of 50 lung cancer patients. Results are presented as mean ± SEM, *p < 0.05, **p < 0.01

To investigate the potential role of POU2F2 in the progression of lung cancer, we first assessed the expression levels of POU2F2 in lung cancer tissues and the adjacent tissues isolated from patients through IHC assays. Importantly, we noticed the high expression levels of POU2F2 in human lung cancer tissues, compared to normal tissues (Fig. 1a). Further through Immunoblot, we found POU2F2 expression was upregulated in lung cancer tissues from three different patients, compared to their corresponding normal tissues (Fig. 1b).

We next explored whether POU2F2 was highly expressed in human lung cancer cell lines compared to normal bronchial epithelial cell lines. Four types of human lung cancer cell lines, including calu-3, calu-6, A549, and H1299, and a normal bronchial epithelial cell line BEAS-2B were used to detect the expression of POU2F2 in these cell lines via Immunoblot assays. Similarly, to the previous results, we noticed POU2F2 was upregulated in human lung cancer cell lines compared to the bronchial epithelial cell line (Fig. 1c).

Subsequently, we detect the effects of POU2F2 on the prognosis of lung cancer patients. A total of 50 patients were classified into two groups, including POU2F2 high (25/50, 50%) or low expression (25/50, 50%) groups, according to the staining intensity of POU2F2 (Table 1). We found there was no significant correlation between POU2F2 expression and the clinical features including patients’ gender (*p* = 0.571), age (*p* = 0.370), histology (*p* = 0.777), and differentiation (*p* = 0.395). Importantly, we found the expression of POU2F2 was obviously correlated with the tumor size (*p* = 0.021), TNM stage (*p* = 0.024), and nodal status (*p* = 0.047) of patients with lung cancer (Table 1).

**Table 1:** Distribution of POU2F2 status in lung cancer according to clinicopathological characteristics

Characteristics	Number of patients	POU2F2 expression		*p* value
		Low (< median)	High (≥ median)	
Number	50	25	25	
Sex				
Male	24	11	13	0.571
Female	26	14	12	
Age (years)				
≥ Mean (50)	33	15	18	0.370
< Mean (50)	17	10	7	
Tumor size				
≥ 3 (cm)	30	11	19	0.021*
< 3 (cm)	20	14	6	
Histology				
Adenocarcinoma	27	13	14	0.777
Squamous cell carcinoma	23	12	11	
TNM stage				
I–II	24	16	8	0.024*
III	26	9	17	
Nodal status				
N0–N1	23	15	8	0.047*
N2–N3	27	10	17	
Differentiation				
Well-moderate	23	13	10	0.395
Poor	27	12	15	

*p < 0.05

Through Kaplan–Meier survival analysis, we found the expression of POU2F2 was significantly correlated with the surviving fraction of lung cancer patients (*p* = 0.0385), suggesting the obvious correlation with prognosis (Fig. 1d). Collectively, we thought POU2F2 was highly expressed in human lung cancer tissues and correlated with patients’ prognosis and clinical features.

### POU2F2 promotes the proliferation, and motility of lung cancer cells in vitro

**Fig. 2:**
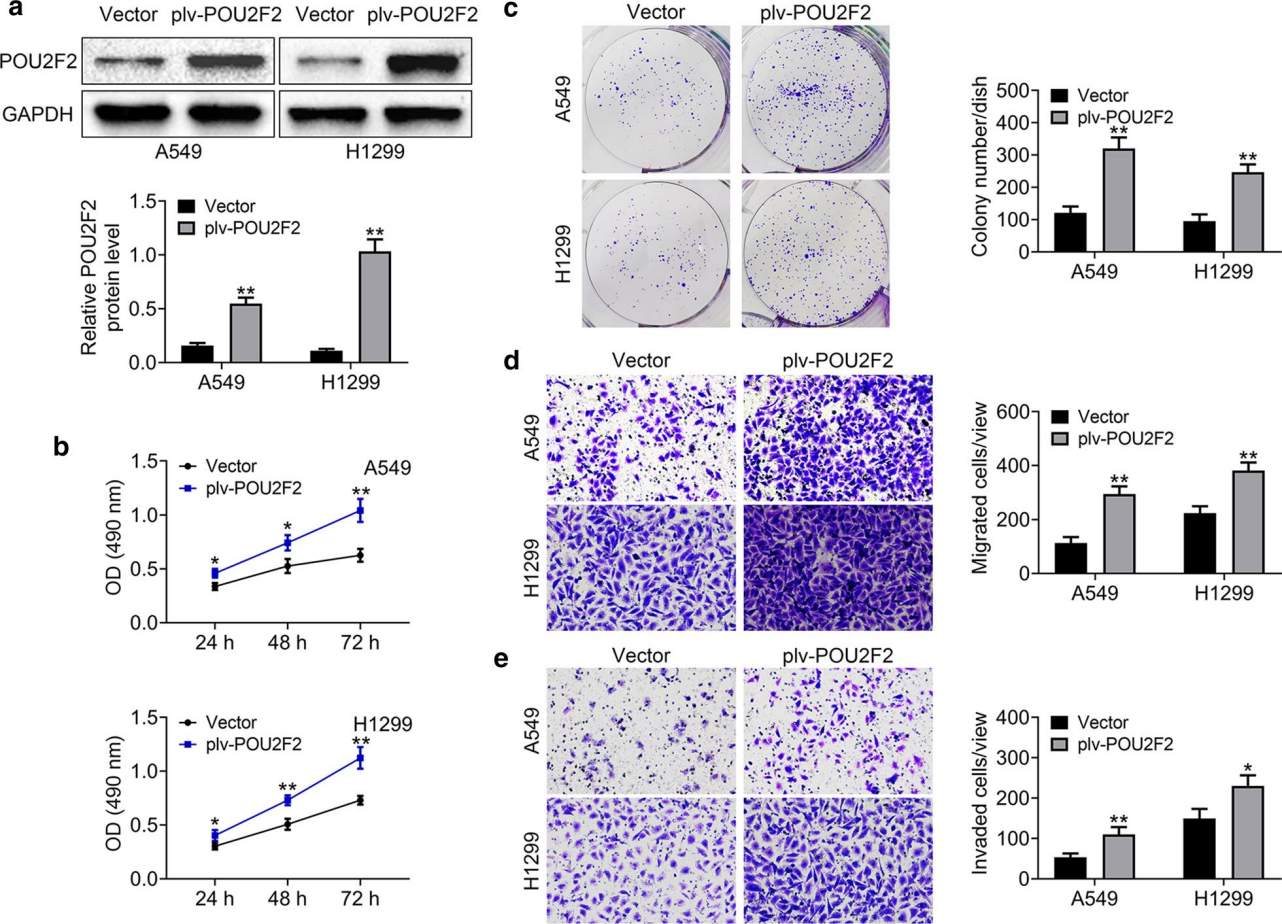
POU2F2 promotes the proliferation, migration, and invasion of lung cancer cells in vitro. **a** Immunoblot assays showed the expression level of POU2F2 in both A549 and H1299 cells transfected with vector or plv-POU2F2 plasmids. These images were analyzed with the Image J (National Institutes of Health, USA). Original image blots are presented in Additional file 3. **b** CCK-8 assays were conducted to detect the effects of POU2F2 overexpression on the proliferation of A549 and H1299 cells, and the OD value at 490 nm wavelength was compared. **c** Colony formation assays were conducted to detect the effects of POU2F2 overexpression on the proliferation of A549 and H1299 cells, and the colony number was calculated. **d** Transwell migration assays were conducted to detect the effects of POU2F2 overexpression on the migration of A549 and H1299 cells, and the OD value at 490 nm wavelength was detected. **e** Transwell invation assays were conducted to detect the effects of POU2F2 overexpression on the invasion of both A549 and H1299 cells, and the OD value at 490 nm wavelength was measured. Results are presented as mean ± SEM, *p < 0.05, **p < 0.01

Since POU2F2 was highly expressed in lung cancer tissues and correlated with the prognosis, we then detected its effects on the progression of lung cancer in vitro. First, plv-POU2F2 plasmids were constructed and transfected into two types of lung cancer cells, including A549 and H1299 cells, respectively, to induce the overexpression of POU2F2 in these cells. Through Immunoblot assays, we confirmed that the transfection of POU2F2 resulted in the upregulation of POU2F2 in both A549 and H1299 cells, compared to the vector groups (Fig. 2a). Both CCK-8 and colony formation assays confirmed that POU2F2 overexpression promoted the proliferation of both A549 and H1299 cells, according to the colony number (Fig. 2b, c). Through transwell-migration assays, we found POU2F2 overexpression stimulated the migration of A549 and H1299 cells through the basement membrane without matrigel (Fig. 2d). Similarly, transwell-invasion assays provided the evidence that the overexpression of POU2F2 led to easier passage of A549 and H1299 cells through the basement membrane coated with 20% matrigel, suggesting the promotion of invasion (Fig. 2e). Therefore, we thought POU2F2 promoted the proliferation, and motility of lung cacner cells in vitro.

### POU2F2 depletion restrained the proliferation, and motility of lung cancer cells

**Fig. 3:**
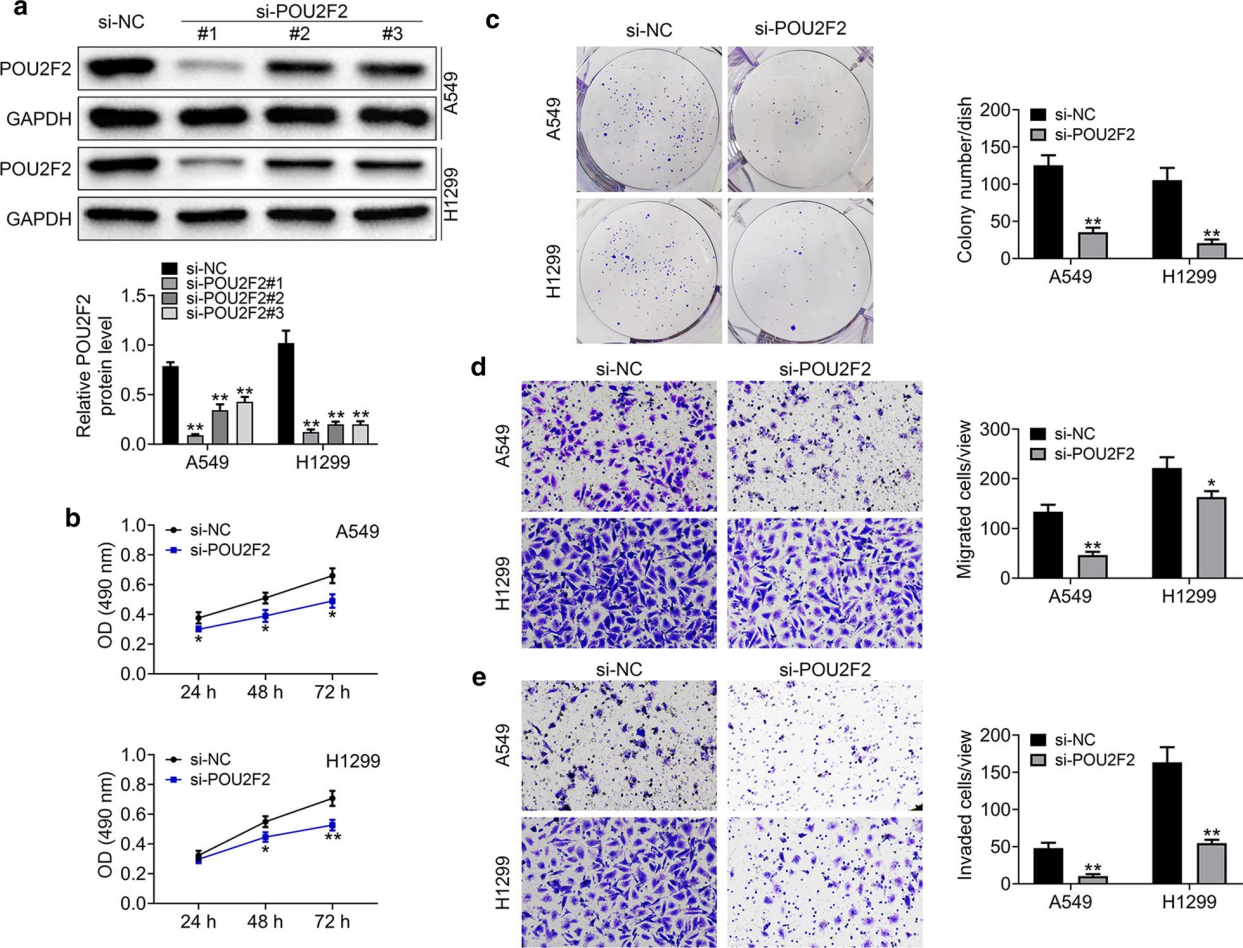
POU2F2 depletion restrained the proliferation, migration, and invasion of lung cancer cells. **a** Immunoblot assays showed the expression level of POU2F2 in both A549 and H1299 cells transfected with control or 3 different siRNAs of POU2F2. These images were analyzed with the Image J (National Institutes of Health, USA). Original image blots are presented in Additional file 4. **b** CCK-8 assays were conducted to detect the effects of POU2F2 depletion on the proliferation of A549 and H1299 cells, and the OD value at 490 nm wavelength was compared. **c** Colony formation assays were conducted to detect the effects of POU2F2 depletion on the proliferation of A549 and H1299 cells, and the colony number was calculated. **d** Transwell migration assays were conducted to detect the effects of POU2F2 depletion on the migration of A549 and H1299 cells, and the OD value at 490 nm wavelength was detected. **e** Transwell invation assays were conducted to detect the effects of POU2F2 depletion on the invasion of both A549 and H1299 cells, and the OD value at 490 nm wavelength was measured. Results are presented as mean ± SEM, *p < 0.05, **p < 0.01

To further confirm the previous conclusion, we used 3 types of siRNAs of POU2F2 to deplete its expression in both A549 and H1299 cells. Through Immunoblot assays, we noticed the transfection of both of these 3 siRNAs could knockdown the expression of POU2F2 in A549 and H1299 cells, respectively (Fig. 3a). Meanwhile, we noticed the si-POU2F2#1 had the highest silent efficiency (Fig. 3a). Therefore, subsequent in vitro experiments were carried out with this siRNA.

Through colony formation assays, we noticed that the depletion of POU2F2 suppressed the proliferation of both A549 and H1299 cells, confirmed by CCK-8 and colony formation assays (Fig. 3b, c). Through transwell-migration assays, the results showed POU2F2 ablation suppressed the migration of A549 and H1299 cells through the basement membrane without matrigel (Fig. 3d). Additionally, the results of transwell-invasion assays indicated the ablation of POU2F2 inhibited A549 and H1299 cells through the basement membrane coated with 20% matrigel, suggesting the inhibition of cell invasion (Fig. 3e). Therefore, POU2F2 depletion could inhibit the proliferation, migration, and invasion of lung cancer cells.

### POU2F2 regulated the expression of AGO1 in lung cancer cells

**Fig. 4:**
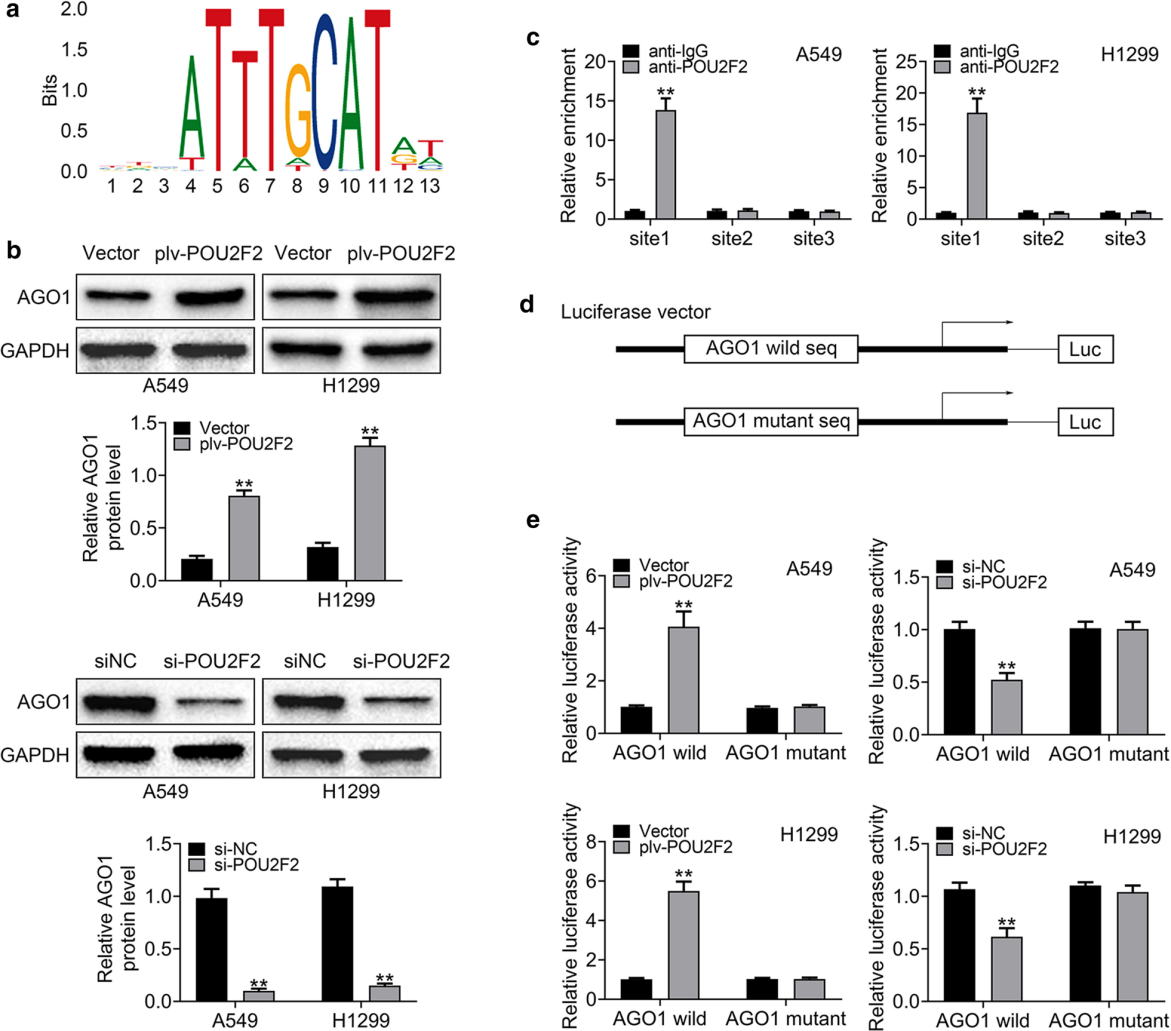
POU2F2 regulated the expression of AGO1 in lung cancer cells. **a** The prediction by Jasper website (http://jaspar.genereg.net) showed POU2F2 could target the sequence of AGO1 promoter regions. **b** Immunoblot assays showed the expression level of POU2F2 in both A549 and H1299 cells transfected with the indicated plasmids or siRNAs. These images were analyzed with the Image J (National Institutes of Health, USA). Original image blots are presented in Additional file 5. **c** qPCR amplification of the anti-IgG or anti-POU2F2 antibody enriched three sites of promoter fragment in A549 and H1299 cells performing CHIP assays. **d**, **e** Luciferase activity of AGO1 wild and mutant in A549 and H1299 cells co-transfected with plv-POU2F2 or plv-vector plasmids was analyzed by luciferase reporter assays. The schematic diagram was shown in **d**, and the results were in **e**

We then investigated the mechanisms underlying POU2F2 promoting the prolifetion, and motility of lung cancer cells in vitro. The effects of AGO1 in the progression of lung cancer has been widely revealed [20, 21]. Through the prediction by Jasper website (http://jaspar.genereg.net), we found POU2F2 could target AGO1 promoter regions, including three different sites (Fig. 4a). The sites included site 1: 1047–1059 (ACAATTTGTATAT), site 2: 35–47 (CTAATTTTTATAT), and site 3: 1041–1053 (TGTATATTCATTC). We further noticed that POU2F2 overexpression promoted the expression of AGO1 in both A549 and H1299 cells in vitro, through Immunoblot assays (Fig. 4b). As a comparison, we found POU2F2 depletion inhibited the expression of AGO1 in two types of lung cancer cells (Fig. 4b). Through CHIP assays, we noticed that POU2F2 could bind to the promoter region, particularly site 1, of AGO1 in both A549 and H1299 cells (Fig. 4c).

Subsequently, luciferase assays were performed. The wildtype and mutant luciferase plasmids were constructed targeted the sequence of site 1, respectively (Fig. 4d). We found POU2F2 overexpression stimulated the luciferase activity of AGO1 in both A549 and H1299 cells (Fig. 4e). However, when the sequence of site 1 was mutant, POU2F2 overexpression could hardly stimulate the luciferase activity of AGO1 in lung cancer cells, further confirming our conclusion (Fig. 4e). As a comparison, the depletion of POU2F2 inhibited the luciferase activity of AGO1 in both A549 and H1299 cells, whereas the AGO1 mutant lacked a similar effect (Fig. 4e). In summary, POU2F2 could promote the expression of AGO1 in lung cancer cells.

### AGO1 knockdown inhibited the proliferation, and motility of lung cancer cells in vitro

**Fig. 5:**
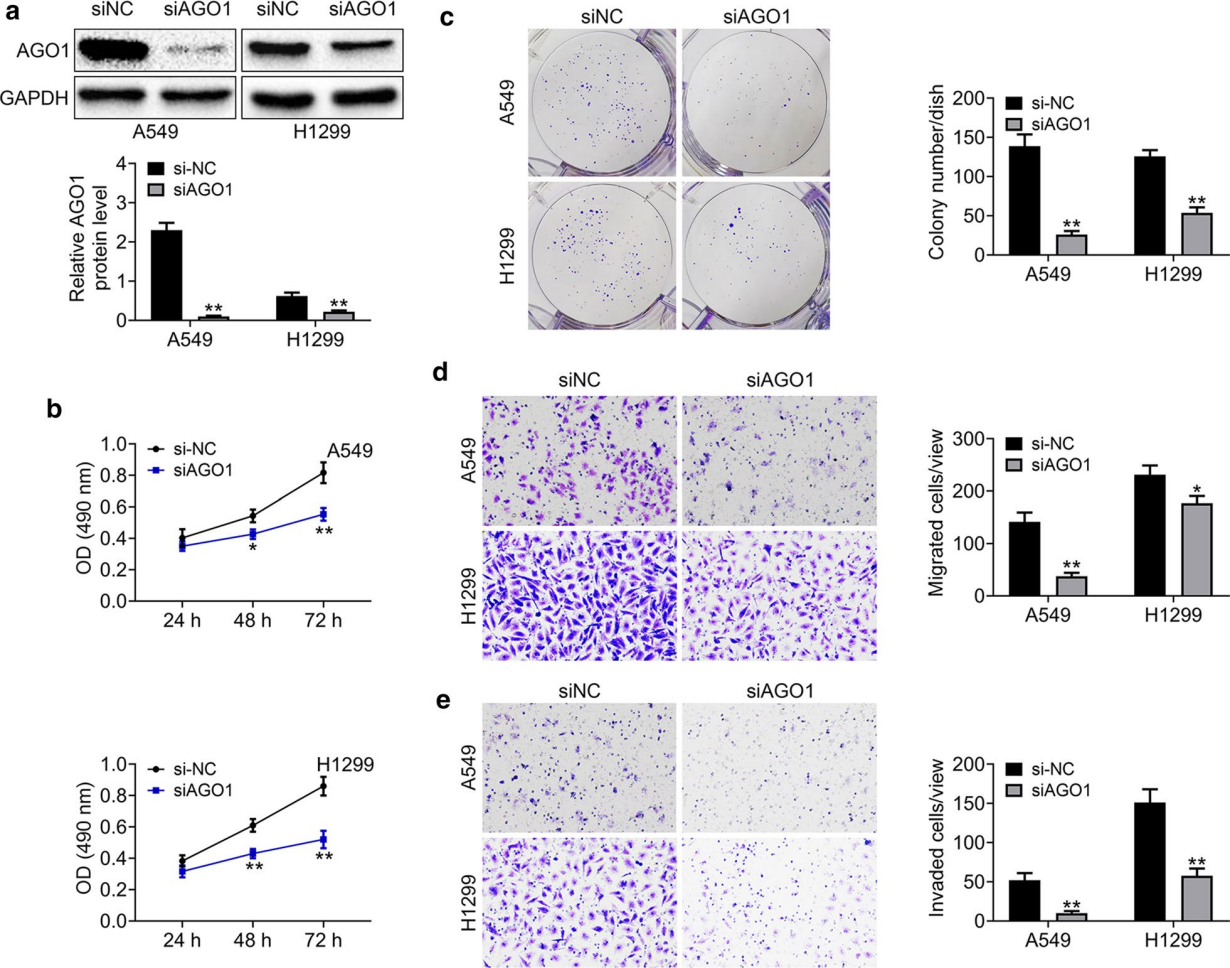
AGO1 knockdown suppressed the proliferation, migration, and invasion of lung cancer cells. **a** Immunoblot assays showed the expression level of AGO1 in both A549 and H1299 cells transfected with control or AGO1 siRNAs. These images were analyzed with the Image J (National Institutes of Health, USA). Original image blots are presented in Additional file 6. **b** CCK-8 assays were conducted to detect the effects of AGO1 depletion on the proliferation of A549 and H1299 cells, and the OD value at 490 nm wavelength was compared. **c** Colony formation assays were conducted to detect the effects of AGO1 depletion on the proliferation of A549 and H1299 cells, and the colony number was shown. **d** Transwell migration assays were conducted to detect the effects of AGO1 depletion on the migration of A549 and H1299 cells, and the OD value at 490 nm wavelength was detected. **e** Transwell invation assays were conducted to detect the effects of AGO1 knockdown on the invasion of both A549 and H1299 cells, and the OD value at 490 nm wavelength was measured. Results are presented as mean ± SEM, *p < 0.05, **p < 0.01

We then investigated the effects of AGO1 on the proliferation, migration, and invasion of lung cancer cells in vitro. A siRNA targeted AGO1 was used and transfected into both A549 and H1299 cells to deplete the expression of AGO1, and the silencing efficiency of the siRNA was confirmed through Immunoblot assays (Fig. 5a). Subsequently, CCK-8 and colony formation assays showed AGO1 ablation suppressed the proliferation of lung cancer cells (Fig. 5b, c). Additionally, the transwell assays provided the evidence that AGO1 knockdown impaired the migration and invasion of both A549 and H1299 cells, respectively (Fig. 5d, e). Collectively, these results showed AGO1 knockdown inhibited the proliferation, and motility of lung cancer cells in vitro.

### POU2F2 promotes the proliferation, and motility of lung cancer cells via targeting AGO1

**Fig. 6:**
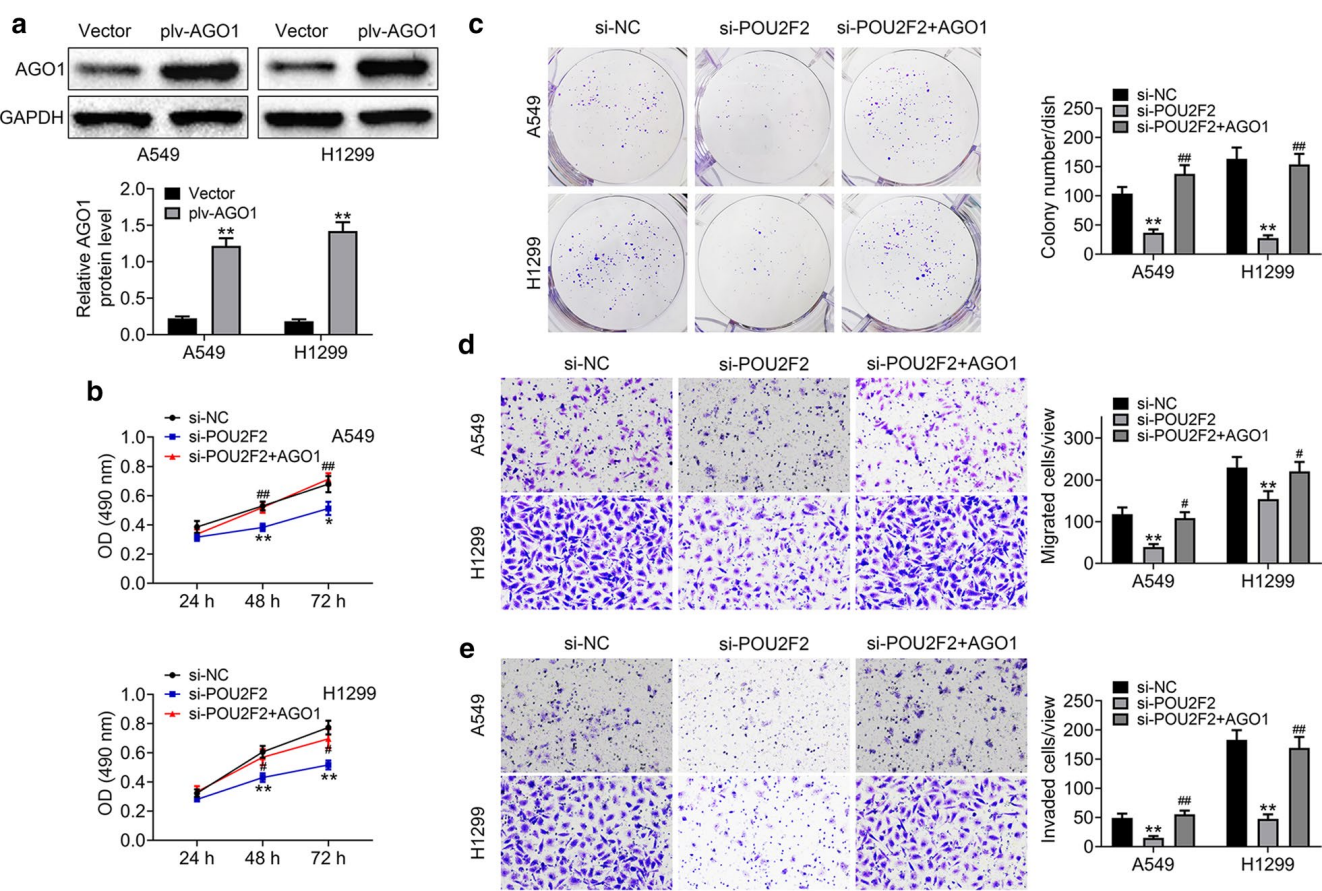
POU2F2 promotes the proliferation, migration, and invasion of lung cancer cells via targeting AGO1. **a** Immunoblot assays showed the expression level of AGO1 in both A549 and H1299 cells transfected with vector or plv-AGO1 plasmids. These images were analyzed with the Image J (National Institutes of Health, USA). Original image blots are presented in Additional file 7. **b** CCK-8 assays were conducted to detect the proliferation capacity of A549 and H1299 cells upon the transfection of the indicated siRNA or plasmids, and the OD value at 490 nm wavelength was detected. **c** Colony formation assays were conducted to detect the proliferation capacity of A549 and H1299 cells upon the transfection of the indicated siRNA or plasmids, and the colony number was shown. **d** Transwell migration assays were conducted to detect the migration degree of A549 and H1299 cells upon the transfection of the indicated siRNA or plasmids, and the OD value at 490 nm wavelength was detected. **e** Transwell invation assays were conducted to detect the invasion degree of both A549 and H1299 cells upon the transfection of the indicated siRNA or plasmids, and the OD value at 490 nm wavelength was measured. Results are presented as mean ± SEM, *p < 0.05, **p < 0.01, ^##^*p* < 0.001

Then we explored whether POU2F2 promoted the proliferation, migration, and invasion of lung cancer cells via AGO1. The plv-AGO1 plasmids were constructed and transfected into both A549 and H1299 cells to induce AGO1 overexpression, which was confirmed by Immunoblot assays (Fig. 6a). Subsequently, we performed rescue assays. Through colony formation assays, we found the depletion of POU2F2 suppressed the proliferation of both A549 and H1299 cells, whereas the overexpression of AGO1 obviously reversed the proliferation defects caused by POU2F2 (Fig. 6b, c). Through transwell-migration assays, we also found the depletion of POU2F2 inhibited the migration of both A549 and H1299 cells, and the overexpression of AGO1 could rescue the inhibition of migration caused by the knockdown of POU2F2 (Fig. 6d). Similarly, transwell-invasion assays showed AGO1 overexpression significantly rescued the invasion impairment after the transfection of POU2F2 siRNAs in both A549 and H1299 cells (Fig. 6e). Therefore, we thought POU2F2 promoted the proliferation, migration, and invasion of lung cancer cells via targeting AGO1.

### POU2F2 promotes tumor growth of lung cancer cells via AGO1 in vivo

**Fig. 7:**
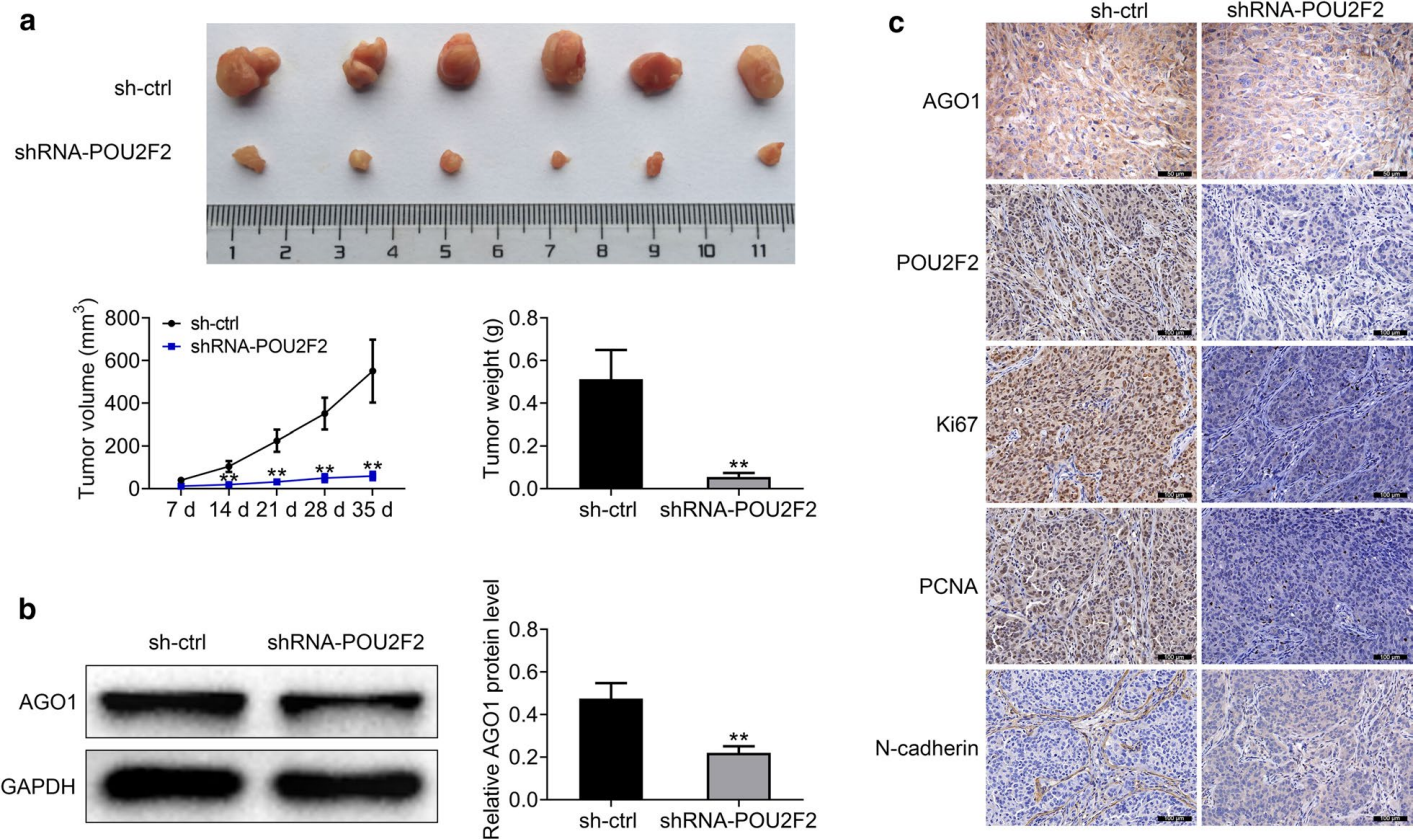
POU2F2 promoted tumor growth of lung cancer cells via AGO1 in mice. **a** Representative photographs of tumors in nude mice formed by A549 cells stably transfected with control or POU2F2 shRNA lentivirus (n = 6 in each group). Volume and weight of tumors from different groups was measured. **b** Immunobolt results showed the expression level of AGO1 in control and POU2F2 knockdown tumor tissues. These images were analyzed with the Image J (National Institutes of Health, USA). Original image blots are presented in Additional file 8. **c** Immunohistochemical results revealed the expression level of the indicated proteins in control and POU2F2-depleted tumor tissues. Results are presented as mean ± SEM, **p < 0.01

Our previous data showed that POU2F2 promoted lung cancer cell proliferation, migration, and invasion in vitro. To further confirm whether POU2F2 ablation could suppress tumor growth, the in vivo assays were performed. A549 cells stably transfected with control or AGO1 shRNA plasmids were injected into nude mice. 1 week later, tumor volume was measured every week. 6 representative tumor samples in each group mice were photographed and exhibited in Fig. 7a. After 35 days, all the tumors were isolated, tumor volume was compared, and the growth curves were calculated. We found the tumors in POU2F2-depleted groups were markedly smaller than control (Fig. 7a). And the tumor weight in POU2F2-depleted groups was also decreased markedly comapred to control (Fig. 7a). Immunoblot assays provided the evidence that the expression of AGO1 in POU2F2-depleted tumor tissues were obvious decrease compared with that in control, consistent with the previous data (Fig. 7b). Through Immunohistochemical assays, we found the depletion of POU2F2 resulted in the decrease of POU2F2 and AGO1 expression, similar to previous results (Fig. 7c). Additionally, we further found POU2F2 ablation suppressed the expression of Ki67, PCNA, and N-cadherin in tumor tissues, suggesting the inhibition of tumor growth (Fig. 7c). Collectively, these results showed that POU2F2 promoted tumor growth of lung cancer cells via AGO1 in mice.

## Discussion

In recent years, multiple advances have been made in the targeted therapy of lung cancer [22]. For different pathological types of lung cancer, there are specific therapeutic targets and the related drugs for use in clinical or in clinical trials [23, 24]. To improve the survival and prognosis of lung cancer patients in the advanced stage, the development of new targeted therapeutic drugs is still urgent requirement [25]. Interestingly, we found a transcription factor, POU2F2, was high expression in human lung cancer tissues and cell lines. Our data further confirmed POU2F2 affected the progression of lung cacner in vitro and in mice. Therefore, POU2F2 could have the potential to serve as a therapeutic target for lung cancer, which needs further study.

As a transcription factor, POU2F2 could bind to the DNA regions and activate the transcription of multiple downstream genes [26]. POU2F2 has extensive effects on several physiological and pathological processes [27]. POU2F2, together with POU2F1, could control the cone photoreceptor production timing in mouse retina [27]. POU2F2 formed a complex with POU2F1 on the iNOS promoter and repressed transcription by interfering with recruitment of RNA PolII by POU2F1 [28]. Next we should detect whether POU2F2 and POU2F1 have synergistic effect in the progress of lung cancer. Another study showed that POU2F2 could cooperate with EBNA1 to promote OriP-dependent transcription [29]. Our data confirmed that POU2F1 transcriptionally activated AGO1 and therefore promoted the progression of lung cancer, and the precise molecular mechanisms and the signaling pathways need further study.

Several previous studies provided the evidence that POU2F2 could serve as a promising therapeutic target and predict the poor prognosis of cancers [16]. POU2F2 predicted the poor prognosis of patients with neuroblastoma and large cell lymphomas [18, 30]. Additionally, POU2F2 was a useful marker for the diagnosis of nodular lymphocyte predominant Hodgkin lymphoma [31]. In this study, we noticed POU2F2 was highly expressed in lung cancer tissues, and importantly, we found POU2F2 affected the progression of lung cancer in vitro and in mice. These studies, together with our findings, comfirmed the critical role of POU2F2 in cancer progression.

Through bioinformatics analysis, we found that POU2F2 could potentially regulate the expression of AGO1, which was verified by further in vitro and in vivo experiments. AGO1 is an argonaute family RNA-binding protein, which is also a component of the miRNA-dependent RNA-induced silencing complex (RISC) [32]. AGO1 could control cell and tissue growth, and affected the progression of multiple types of cancers, such as breast cancer and liver cancer [21, 33]. AGO1 could influence the prognosis of hepatocellular carcinoma (HCC) through TGF-β pathway [21]. However, its possible role in lung cancer was also unclear. We here found the involvement of AGO1 in lung cancer progression, and further confirmed that POU2F2 regulated lung cancer progression via AGO1. Our data suggested that POU2F2-AGO1 axis might serve as a molecular target for lung cancer treatment.

## Conclusions

In summary, we noticed the high POU2F2 levels in human lung cancer tissues and cell lines. POU2F2 expression correlated with the prognosis and clinical features of lung cancer patients. We further demonstrated POU2F2 could promote the proliferation and motility of lung cancer cells through transcriptionally activating AGO1, and fascinate tumor growth of lung cancer cells in mice. We therefore thought POU2F2 as a lung cancer target.

## Supplementary Information

**Additional file 1.** The original WB image in figure1B.

**Additional file 2.** The original WB image in figure1C.

**Additional file 3.** The original WB image in figure 2A.

**Additional file 4.** The original WB image in figure 3A.

**Additional file 5.** The original WB image in figure 4B.

**Additional file 6.** The original WB image in figure 5B.

**Additional file 7.** The original WB image in figure 6A.

**Additional file 8.** The original WB image in figure 7B.

## Data Availability

All data generated or analyzed during this study are included in this published article.

## Abbreviations

POU2F2: POU domain class 2 transcription factor 2
IHC: Immunohistochemical
OCT2: Octamer-binding protein 2
