# Supplementary figures and images for: POU2F2 promotes the proliferation and motility of lung cancer cells by activating AGO1
Source: BMC Pulm Med. 2021 Apr 8;21:117. doi: 10.1186/s12890-021-01476-9 (PMC8034198; doi:10.1186/s12890-021-01476-9)

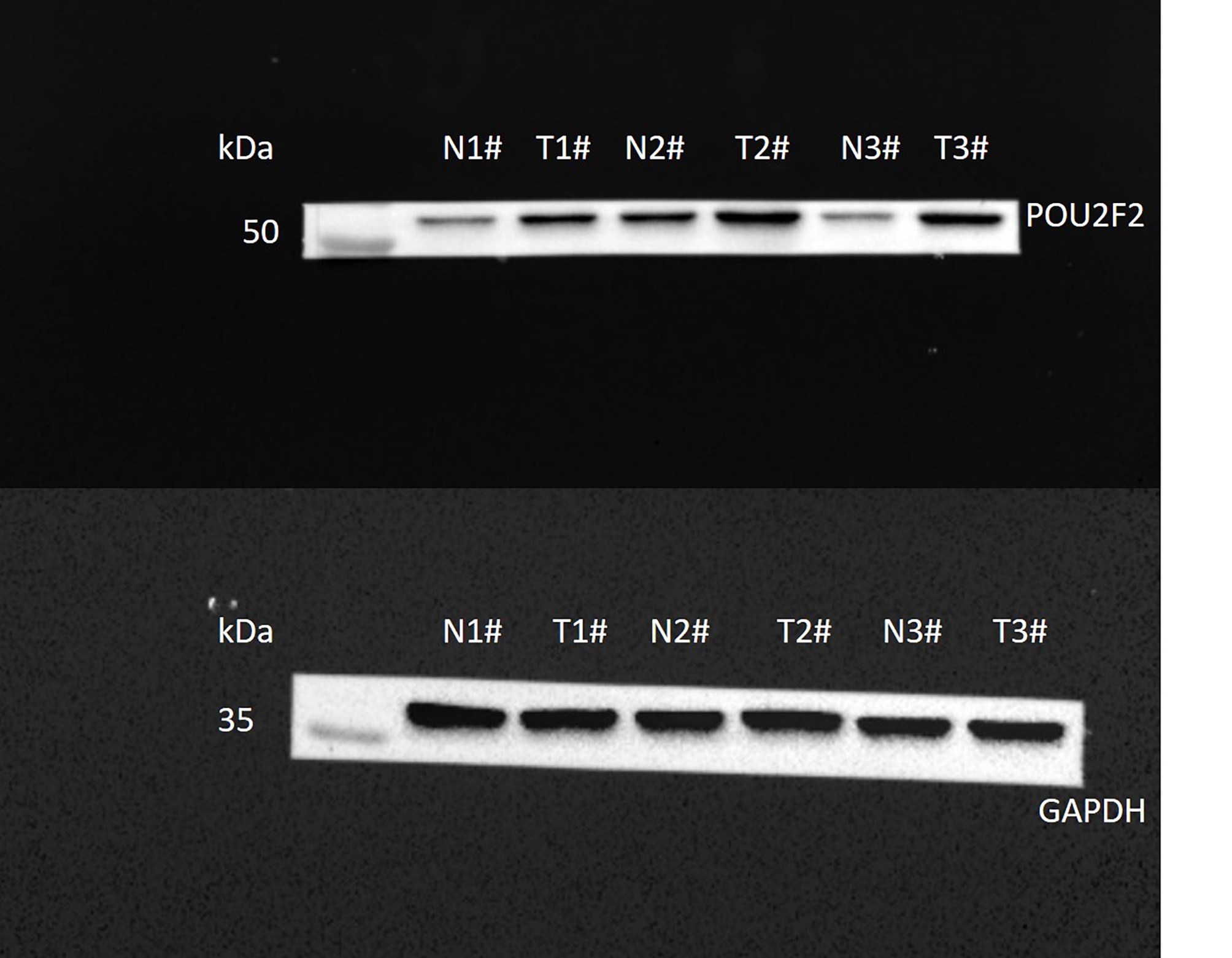

Supplement: Supplementary file 1 — Additional file 1. The original WB image in figure1B. [file 12890_2021_1476_MOESM1_ESM.jpg]

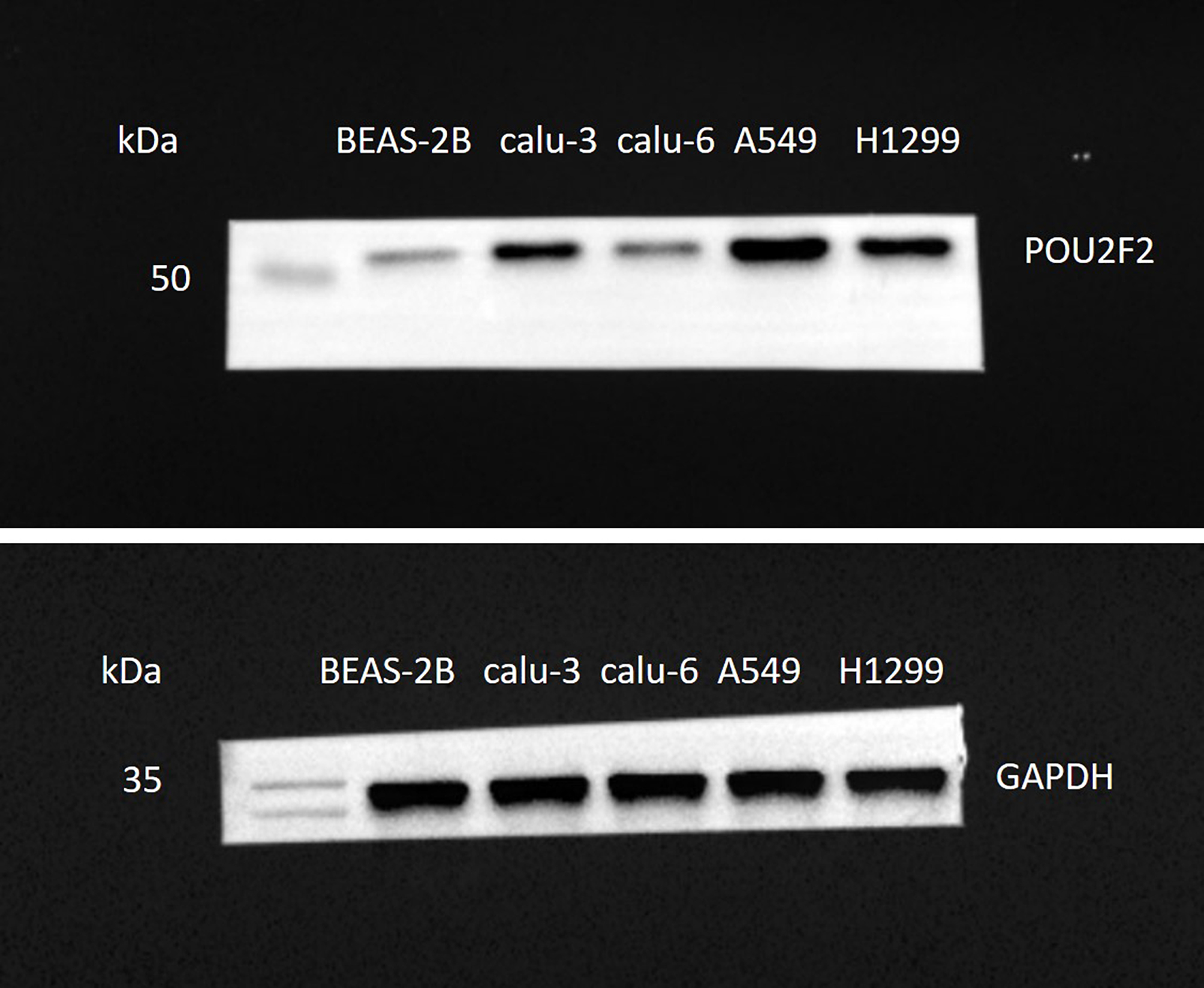

Supplement: Supplementary file 2 — Additional file 2. The original WB image in figure1C. [file 12890_2021_1476_MOESM2_ESM.jpg]

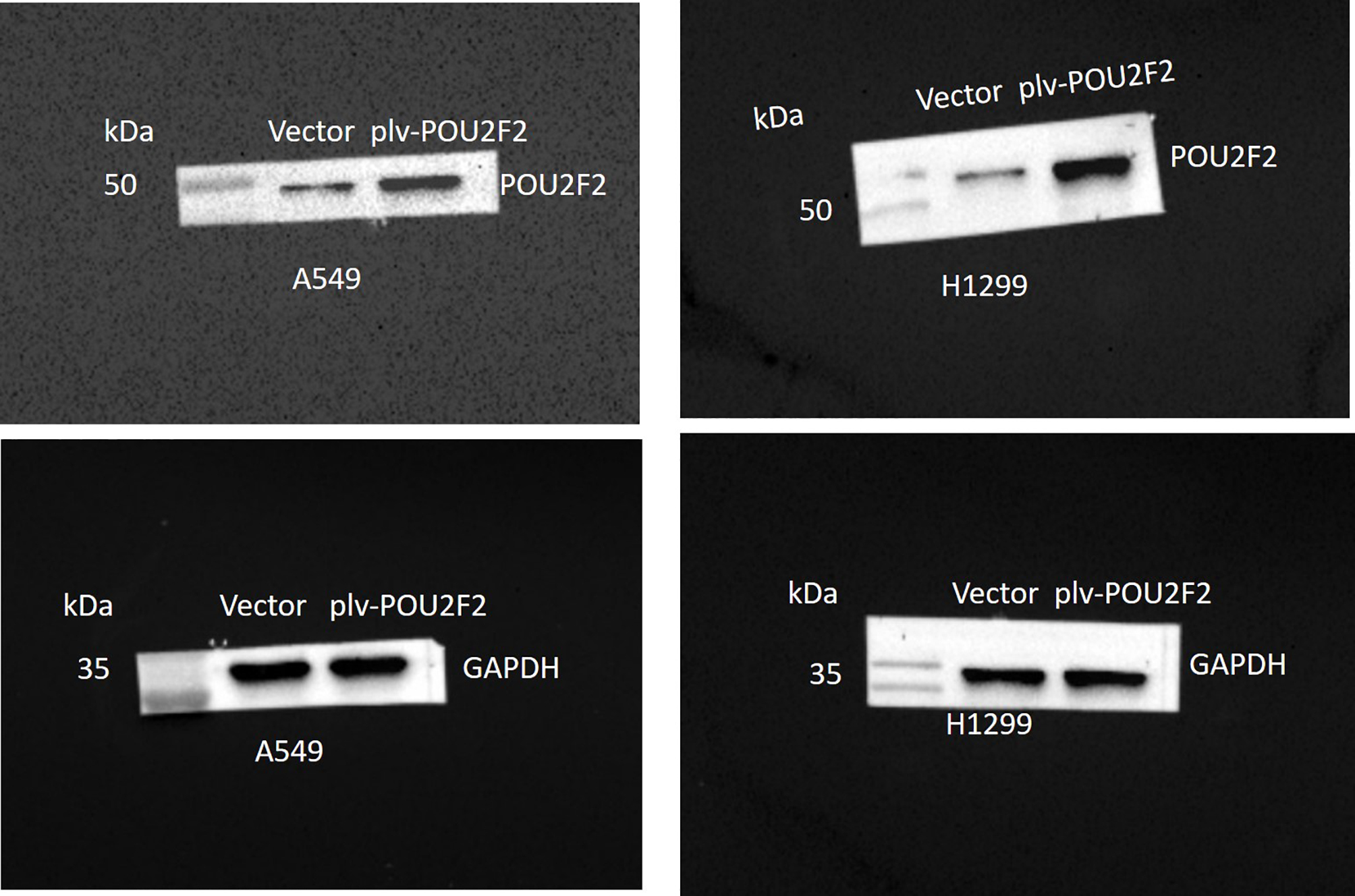

Supplement: Supplementary file 3 — Additional file 3. The original WB image in figure 2A. [file 12890_2021_1476_MOESM3_ESM.jpg]

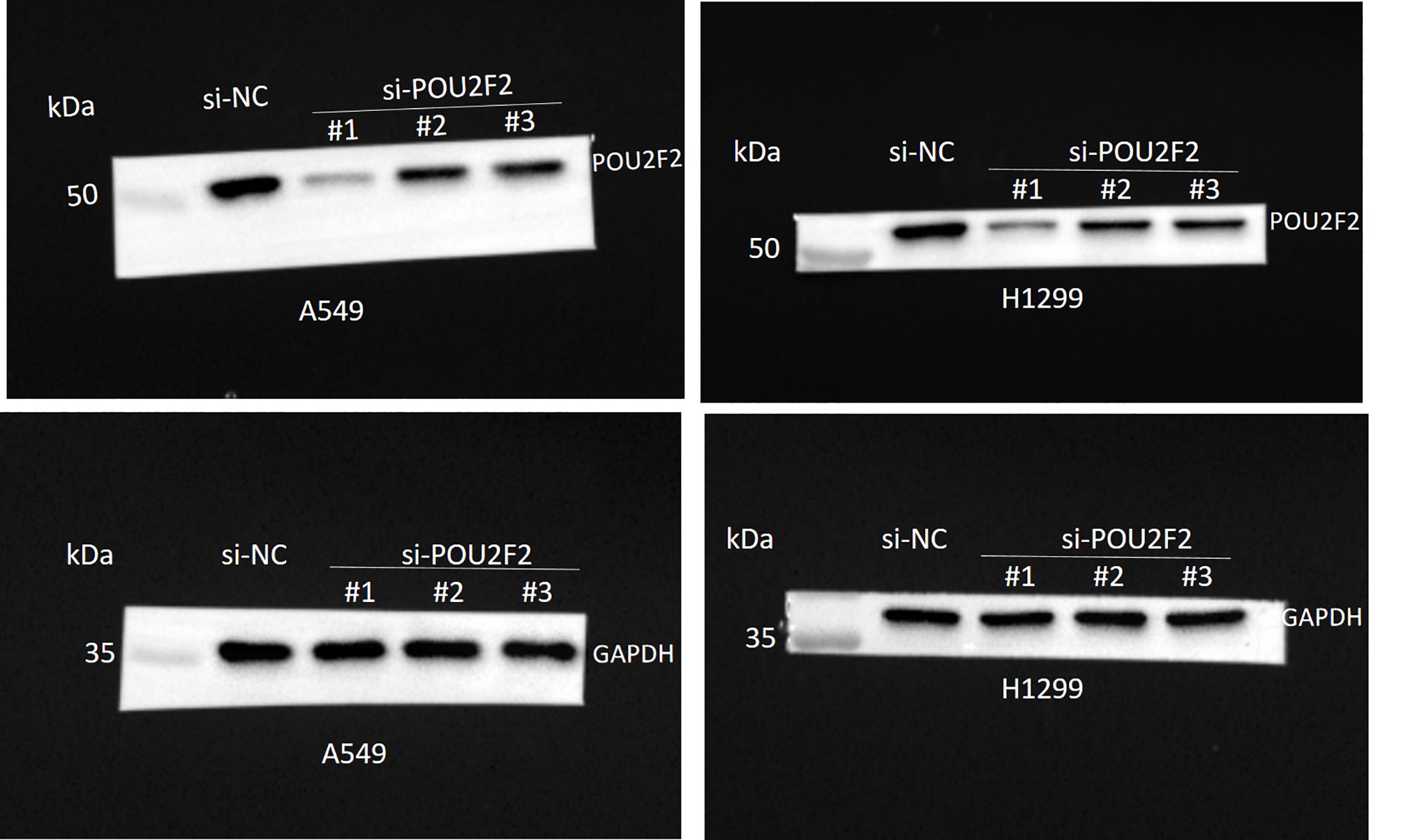

Supplement: Supplementary file 4 — Additional file 4. The original WB image in figure 3A. [file 12890_2021_1476_MOESM4_ESM.jpg]

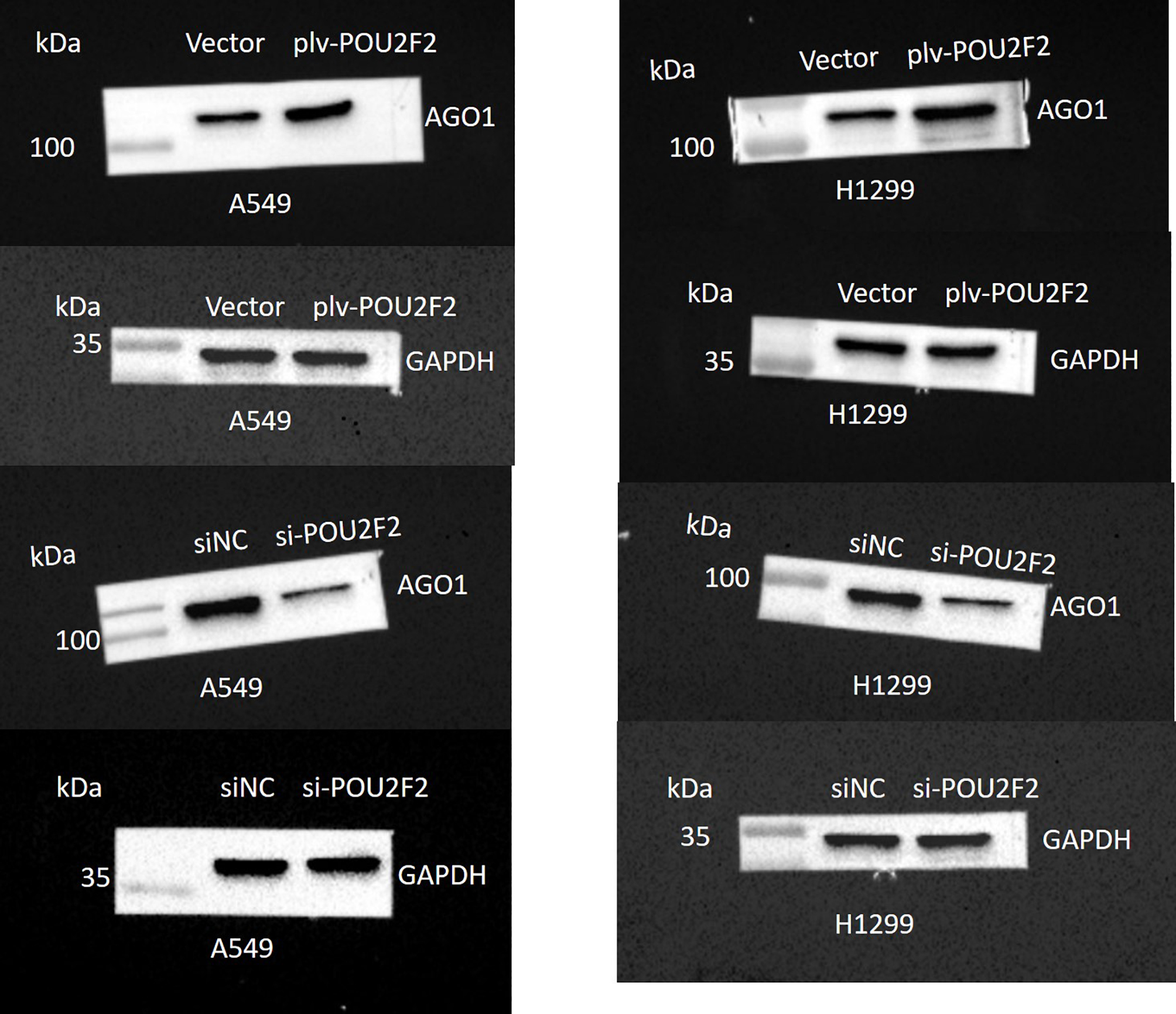

Supplement: Supplementary file 5 — Additional file 5. The original WB image in figure 4B. [file 12890_2021_1476_MOESM5_ESM.jpg]

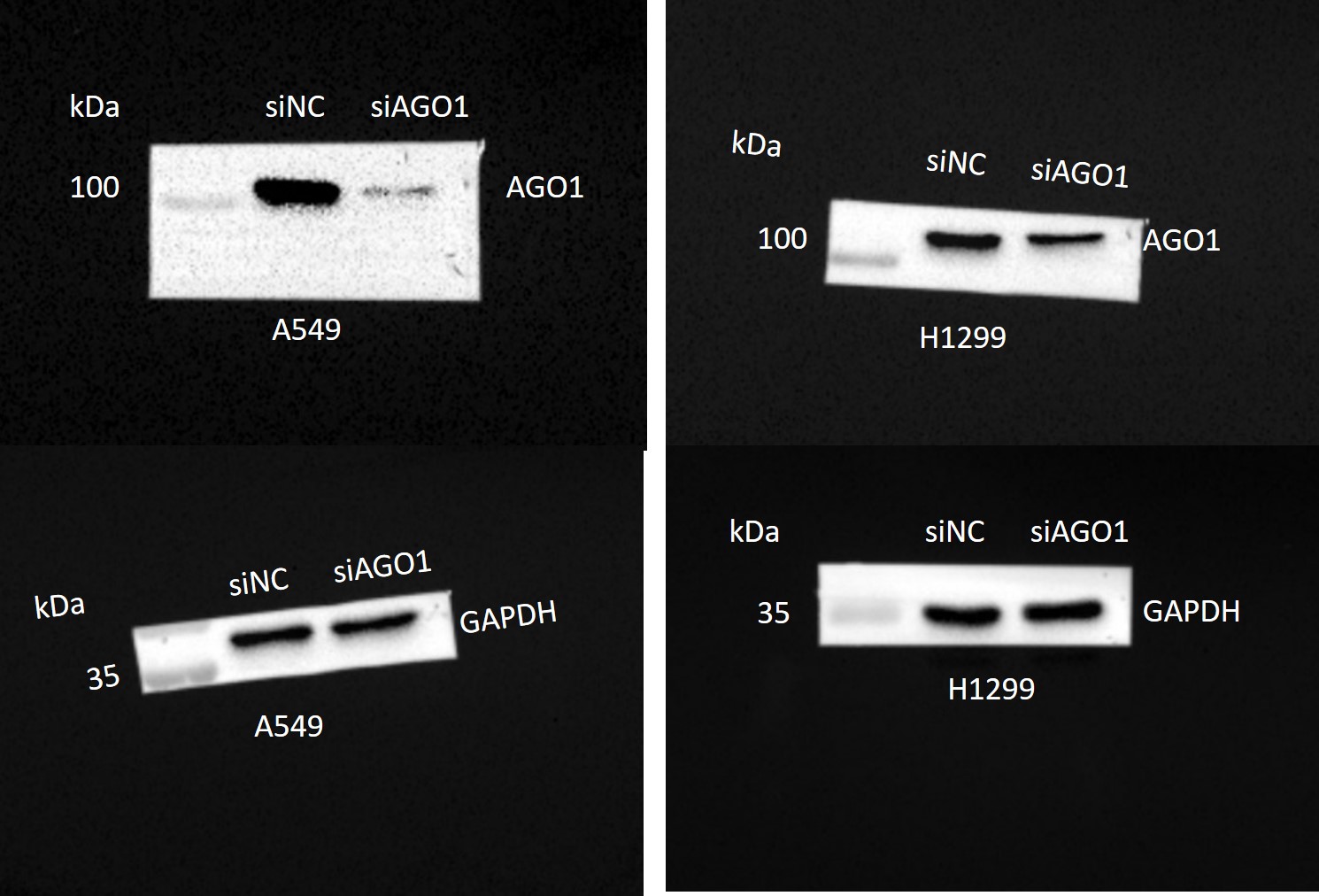

Supplement: Supplementary file 6 — Additional file 6. The original WB image in figure 5B. [file 12890_2021_1476_MOESM6_ESM.jpg]

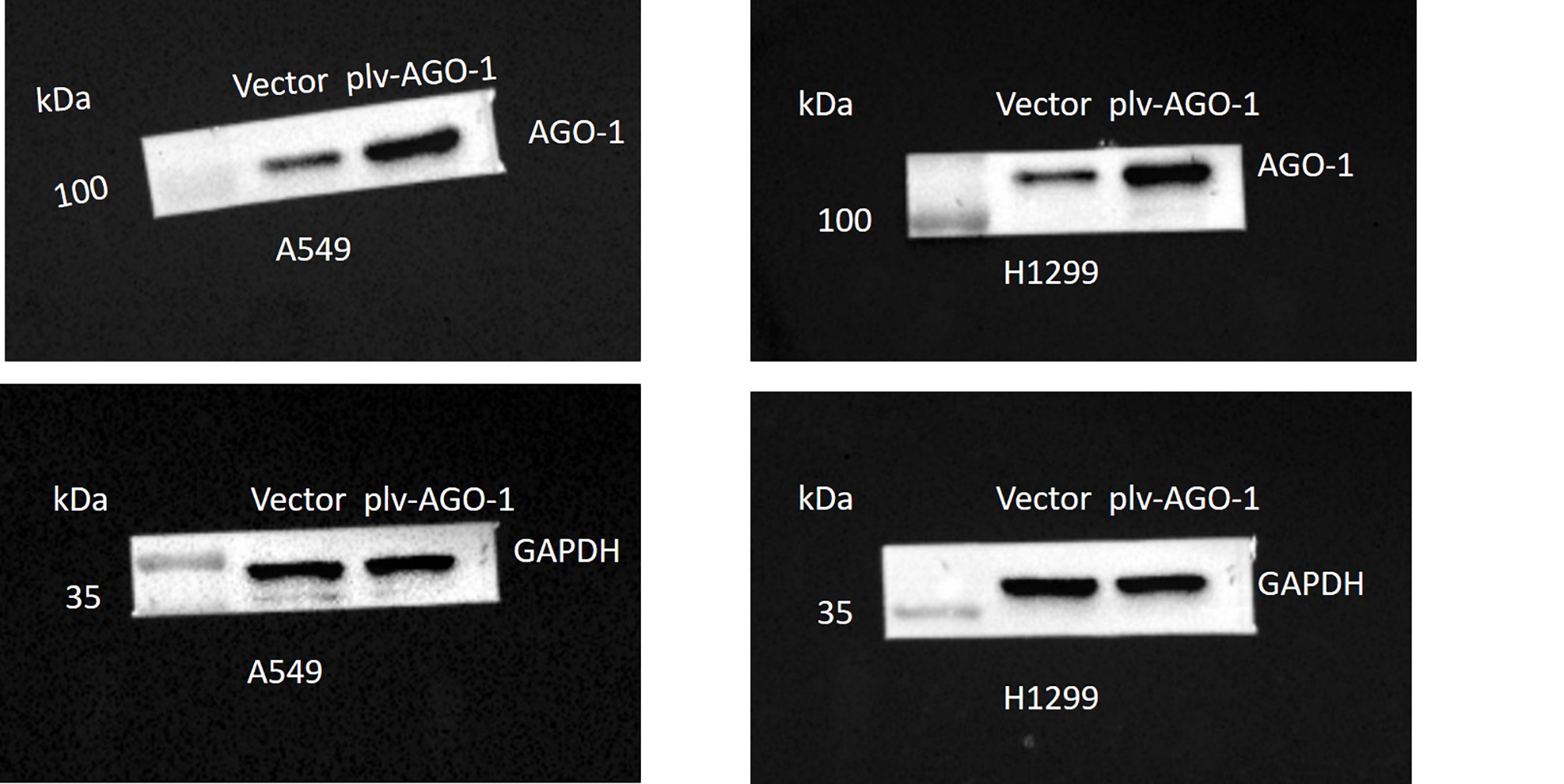

Supplement: Supplementary file 7 — Additional file 7. The original WB image in figure 6A. [file 12890_2021_1476_MOESM7_ESM.jpg]

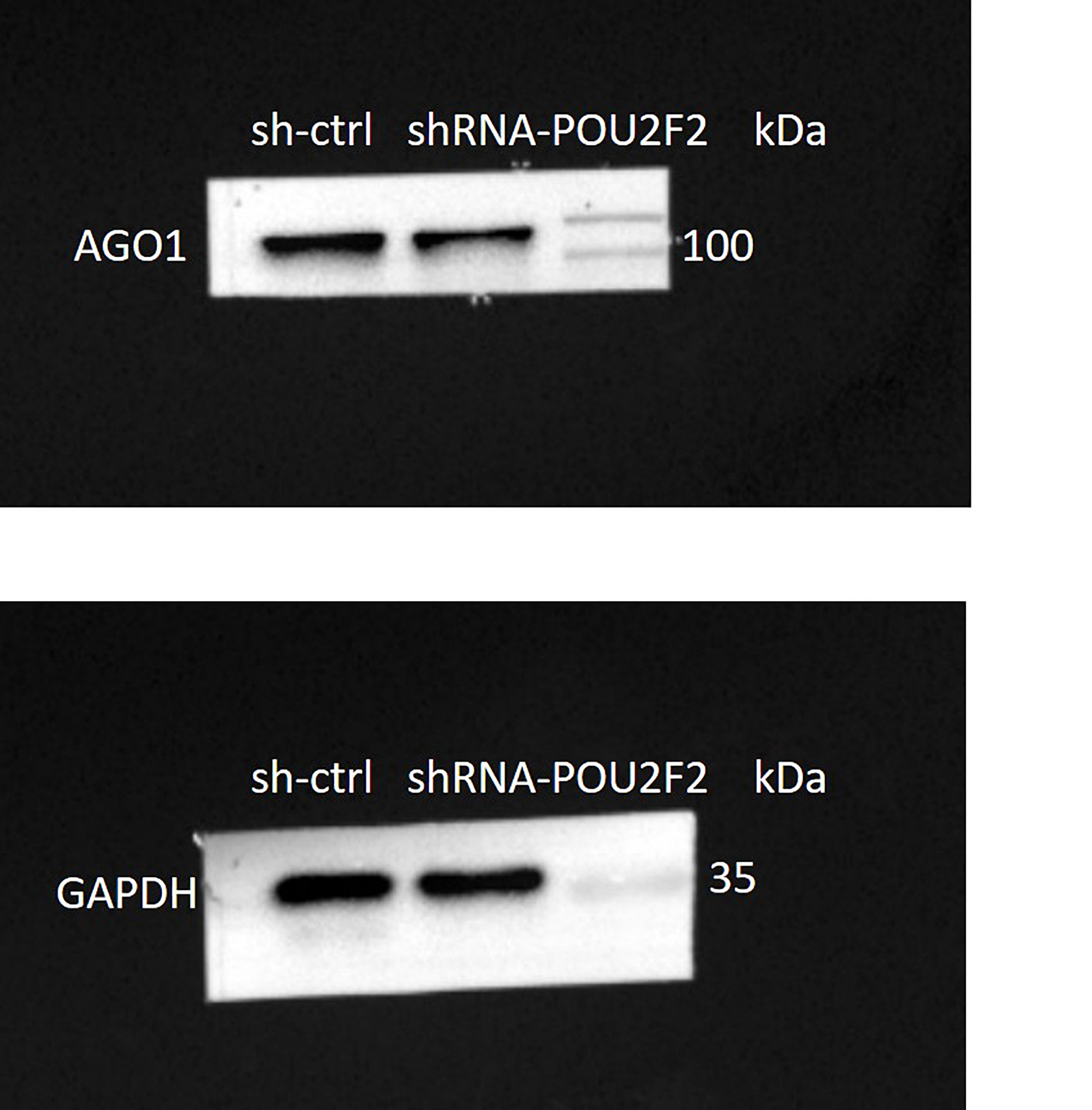

Supplement: Supplementary file 8 — Additional file 8. The original WB image in figure 7B. [file 12890_2021_1476_MOESM8_ESM.jpg]
